# Liquid Metal Based Nano-Composites for Printable Stretchable Electronics

**DOI:** 10.3390/s22072516

**Published:** 2022-03-25

**Authors:** Dan Xu, Jinwei Cao, Fei Liu, Shengbo Zou, Wenjuan Lei, Yuanzhao Wu, Yiwei Liu, Jie Shang, Run-Wei Li

**Affiliations:** 1 CAS Key Laboratory of Magnetic Materials and Devices, Ningbo Institute of Materials Technology and Engineering, Chinese Academy of Sciences, Ningbo 315201, China; xd@nimte.ac.cn (D.X.); jinwei.cao@nottingham.edu.cn (J.C.); liufei2021@nimte.ac.cn (F.L.); zoushengbo@nimte.ac.cn (S.Z.); leiwenjuan@nimte.ac.cn (W.L.); wuyz@nimte.ac.cn (Y.W.); liuyw@nimte.ac.cn (Y.L.); 2 Zhejiang Province Key Laboratory of Magnetic Materials and Application Technology, Ningbo Institute of Materials Technology and Engineering, Chinese Academy of Sciences, Ningbo 315201, China; 3 College of Materials Science and Opto-Electronic Technology, University of Chinese Academy of Sciences, Beijing 100049, China; 4 New Materials Institute, Department of Mechanical, Materials and Manufacturing Engineering, University of Nottingham Ningbo, Ningbo 315100, China

**Keywords:** liquid metal, gallium, composites, stretchable electronics, printable

## Abstract

Liquid metal (LM) has attracted prominent attention for stretchable and elastic electronics applications due to its exceptional fluidity and conductivity at room temperature. Despite progress in this field, a great disparity remains between material fabrication and practical applications on account of the high surface tension and unavoidable oxidation of LM. Here, the composition and nanolization of liquid metal can be envisioned as effective solutions to the processibility–performance dilemma caused by high surface tension. This review aims to summarize the strategies for the fabrication, processing, and application of LM-based nano-composites. The intrinsic mechanism and superiority of the composition method will further extend the capabilities of printable ink. Recent applications of LM-based nano-composites in printing are also provided to guide the large-scale production of stretchable electronics.

## 1. Introduction

Stretchable and elastic electronics have consistently been anticipated to realize sensing, monitoring, diagnosing, and functionalizing on human–machine interfaces [1]. Materials are the cornerstone of stretchable electronics, and have been rapidly innovated to integrate electronics into everyday life; this is particularly the case of stretchable conductive materials [2]. Liquid metal (LM), a common choice for conductive materials, possesses excellent deformability and durability, exhibiting promising potential in eliminating the Young’s modulus mismatch in rigid and flexible interfaces [3]. Gallium (Ga), and its eutectic alloys with indium (In) and tin (Sn), display a lower melting point (typically <300 °C), high vapor pressure, and high surface tension, which clearly shows the combination characteristics of metals and fluids. More importantly, LM is widely applied in stretchable electronics due to its supreme electrical conductivity (3.4 $\times \;10^{6}$ S $m^{-1}$) and thermal conductivity (8~82 W $m^{-1}\;K^{-1}$) [4]. Researchers have found that liquid metals have characteristics relating to multiple external stimuli responses, such as temperature, mechanical force, chemical environment, electric field, dielectric field, magnetic field, and even light [5]. As a result of innovative design principles and processing methods, LM has successfully generated many application prospects, e.g., stretchable sensing systems [6,7,8], electrodes [9,10], actuators [11,12], thermoelectric wearables [13], and energy storage systems [14,15].

**Figure 1:**
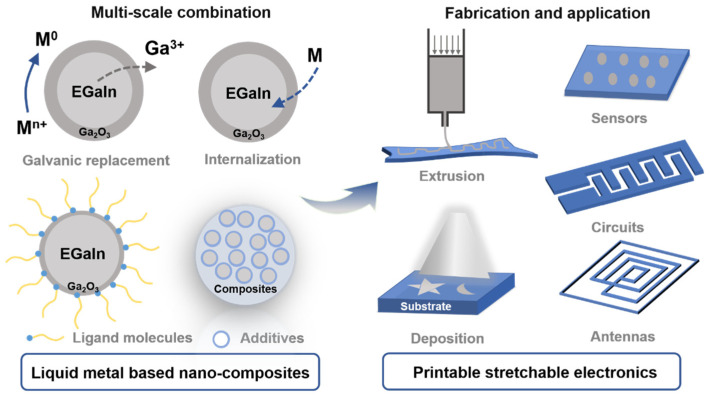
Schematic illustration of liquid metal-based nano-composites and the application of printable stretchable electronics.

Printing technology has many advantages, especially for large-scale and rapid preparation of electronics. Compared to other pattern technologies, such as lithography or vacuum-based processes (e.g., evaporation), which typically require cumbersome and rigorous processes, printing technology applied to electronics usually requires one-step manufacturing and has a low cost. The main issues relate to the high-performance conductive ink and the high-resolution and high-throughput printing. To date, gallium and its alloys have been found to exhibit natural fluidity at room temperature and high conductivity, and are known to be desirable choices for preparing electronic inks. Nevertheless, it still remains an on-going challenge to directly process or pattern LM materials [16]. For example, due to its high surface tension, LM is maintained as droplets or particles in most cases [17], which leads to the weak interface compatibility between liquid metal and substrates. The poor substrate adaptability of LM materials causes an obvious mismatch between the actual design pattern and a loss in the printing speed, and may damage the printed products. Many approaches have been proposed to improve selective wettability during printing, including the pre-treatment and pre-pattern on substrate materials [18,19]. However, due to the inconvenient process, it is difficult for selective printing technology to be generalized in large-scale production. Recently, from a material standpoint, the multi-scale (from micro to nano) combination [20] of other materials with LM (e.g., the composites of metals [21], magnetic materials [22], elastomers [23], and 2D materials [22,24]) has become a popular approach to further enrich and improve its functions, as shown in Figure 1. Enhanced by compositing, these liquid metal-based nano-composites exhibit sufficient capabilities to meet the requirements of printable stretchable electronics. More specifically, the most common composite that consists of LM and its intrinsic oxidation layer enables a rheological property modification of LM, which enhances the interfacial and intermetallic wettability [25]. Through this compounding method, the variety of flowable metals has been fully extended. In addition, on the basis phase of LM oxide, some rigid metals with high conductivity (Ag [26], Au [27], Cu [28]), magnetic properties (Fe [29], Ni [30]), and thermal conductivity have been innovatively introduced to improve the corresponding properties. The resulting materials display properties that more closely resemble those of semifluid sticky ink, with low surface tension and high viscosity. The semifluid LM paste [31] can be easily attached to various substrates, which may enable versatile approaches (such as inkjet and screen-printing technology) for direct patterning of the liquid metal.

Although the oxides can change the material composition to improve interfacial wetting, the intrinsically fragile oxide shell (usually 0.7–3 nm) [32] stabilized LM particles are susceptible to rupture or leakage under strain or pressure stimuli [33,34]. To address this concern, surface ligand modification is potential solution to enable mechanical property and chemical function enhancement of the oxide layer. The LM–ligand molecule composite systems present a stable physical status in dispersion, storage, and solution processing [35]. Printable inks composed of liquid metal nanoparticles retain unique and desirable material properties suitable for high-resolution printed electronics. Furthermore, they can also be exploited as surface intermediates to initiate free radical polymerization [36,37], atom transfer radical polymerization [38], and rapidly gelling [39]. In summary, the LM-based nano-composites have exceptional electrical properties, infinite deformation, and good dispersibility in elastomer matrix, thus promising widespread applications in stretchable conductors and multi-mode sensing systems [40].

The current review mainly focuses on the fabrication, combination mechanism, and subsequent application of Ga-based liquid metal composites in printable stretchable electronics. To provide an in-depth and comprehensive understanding of recent works, this review elaborates the characteristics and advantages of each composite in detail. Due to the properties provided by multiple additives, the LM nano-composites can be fully utilized in the application of printable stretchable electronics, benefiting from their stable dispersion and sufficient interactions with substrates. Correspondingly, different printing technologies are generalized into the two categories of contacting (mask-based) and non-contacting (nozzle-based) modes. After providing insights into the application of printable stretchable electronics, the key challenges and future direction are discussed from the perspectives of material improvement and device performance optimization.

## 2. Liquid Metal-Based Nano-Composites

The original bulk LM trends to maintain a high surface tension, which is not compatible with various advanced additive processing technologies (such as inkjet, aerosol printing, and direct writing). Basically, the interaction at the substrate–ink interface dominates the entire process of printing, storage, and application. To reduce the surface tension of LM, a well-acknowledged strategy of size nanolization has been proposed to realize a large surface area of the oxide layer, which serves as a stabilizer for LM nanoparticles or LM–additive interfaces. The principle of surface tension reduction can be attributed to the reduction in particle size, and the introduction of oxides or other components. Based on this size effect of micro/nano materials and the new function introduced by additives, applications of the LM nano-composites have been tremendously expanded. The size of the liquid metal particles is related to the stability of the printing process and the line width of electronics. Overall, the LM-based nano-composites can be described as material systems in which LM alloys such as EGaIn or Galinstan are suspended as nanoscale droplets in other continuous phases. The earliest printed materials are all conductors, and LMs having high conductivity are often employed in the form of the LM and other metal materials in actual processing. The metal nanoparticles are expected to further improve the conductivity of LM ink, whereas the advantage of elastomers as composites is that encapsulation steps for subsequent printed devices can be eliminated. The key to obtaining LM nano-composites is to use a series of approaches to combine LM and other materials through the interface, or full recombination to obtain high-performance printable nano-inks.

**Figure 2:**
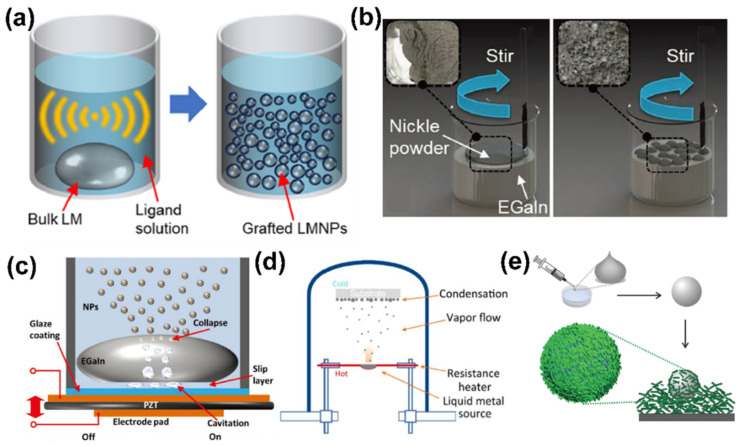
Basic synthesis methods of liquid metal-based nano-composites. (**a**) Ultrasound. Reprinted with permission from Ref. [41]. Copyright 2020 MDPI. (**b**) Shear mixing. Reprinted with permission from Ref. [43] Copyright 2018 John Wiley and Sons. (**c**) Nebulization. Reprinted with permission from Ref. [45] Copyright 2018 John Wiley and Sons. (**d**) Physical vapor deposition. Reprinted with permission from Ref. [46] Copyright 2018 Elsevier. (**e**) Surface rolling coating. Reprinted with permission from Ref. [47] Copyright 2017 Royal Society of Chemistry.

At present, there are many methods for the preparation of LM-based macro- to nano-composites. Ultrasonic treatment [41] (Figure 2a), the most common method for particle nanolization, can shrink bulk LM to nano-sized particles by generating high mechanical energy waves, which can simultaneously in situ functionalize the surface with ligands, polymer, or organic or inorganic materials [42]. Ultrasound can not only disperse droplets, but also reaggregate nanoparticles. The LM-based nanoparticles have a low freezing point under certain conditions and can maintain a liquid phase in a wider temperature range, although there are unknown effects or physical properties. Furthermore, for facile mechanical treatment and scalable production, a shear mixing method [43] (Figure 2b) was also developed to fabricate the nano/micro-sized LM particles. In this process, the secondary oxide shell is formed continuously and prevents the coalescing of LM, and is particularly utilized to introduce intrinsically insoluble additives (magnetic powder, silicone oil and nano-clay) [44]. Structural and functional composites can be generally described according to the specific application. For example, the LM droplets encapsulated in polymer can systemically realize structural separation and performance transition (conductivity and dielectric). In addition, the nebulization [45] (Figure 2c) and physical vapor deposition [46] (Figure 2d) of LM enables nanoparticles with a uniform size and better sphericity than those using ultrasonic treatment. It is also noteworthy to mention that micro-LM particles can be easily fabricated by injecting the LM through a syringe and rolling it on the target materials [47] (Figure 2e). LM has the characteristic of isolating from the surrounding environment, and attaches to sticky materials during the extrusion process.

### 2.1. Liquid Metal-Oxide/Metal Composites

**Figure 3:**
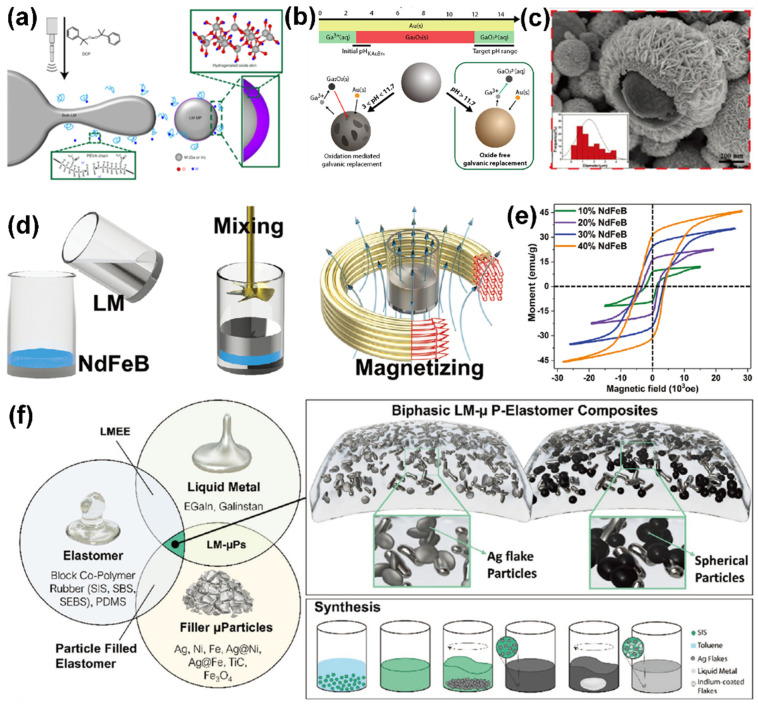
Liquid metal-oxide/metal composites. (**a**) H-doped LM. Reprinted with permission from Ref. [48] Copyright 2021 Springer Nature. (**b**) Au-coated LM. Reprinted with permission from Ref. [51] Copyright 2018 American Chemical Society (**c**) Ag-coated LM. Reprinted with permission from Ref. [26] Copyright 2020 John Wiley and Sons (**d**) Illustration of the FM-LM preparation process. (**e**) Magnetization hysteresis loops of FM-LM under different NdFeB weight fractions. Reprinted with permission from Ref. [54] Copyright 2020 John Wiley and Sons. (**f**) The constitutional materials and synthesis of binary and trinary LM composites. Reprinted with permission from Ref. [55] Copyright 2022 John Wiley and Sons.

LM is known to have an easily functionalized and designable interface, and the spontaneously formed LM oxide layer helps prevent coalescence of LM, which also prevents effective electrical connections between the dispersed particles. The outer shell of these metal oxides severely limits the electrical performance of LM-based printed electronics. In order to obtain conductive LM particles, researchers have made tremendous efforts regarding the composition of the shell, including oxide layer modification and metal coating. Veerapandian et al. revealed that hydrogen doping (increasing defect concentration) introduced by the ultrasonication in aliphatic polymers [48] (Figure 3a) enables the passivate oxide skin with high conductivity and deformability. The printed circuit line with ink made by the H-doped MPs possesses a conductivity (25,000 S cm^−1^) similar even to that of the bulk LM. In addition, another easy-to-understand method exists to eliminate the electrical limitation by coating highly conductive metal (e.g., Pt [49]) on LM particles, which is also known as the galvanic replacement reaction [50]. The metal coating is available through the ultrasonication of LM in the solution with the metal ion precursor (e.g., KAuBr_4_ [27]). It is worth mentioning that the pH plays significant role in the etching effect of the oxide shell. For instance, the composition and morphology of LM particles with a Cu_x_O layer would change massively upon a slight variation in pH [51] (Figure 3b). In addition, Zheng et al. obtained core–shell structural particles (Ag@LMPs) that exhibit excellent initial conductivity (8.0 Ω sq^−1^) by wet-chemical deposition [26]. Specifically, a silver mirror reaction occurs in the solution consisting of precipitants, metal salts, and LMP dispersion (glucose and Ag(NH_3_)_2_OH). Shown in Figure 3c, the porous lamination and heterogeneous structures (thickness from 55 to 259 nm) show its high conductivity. Furthermore, the physical vapor deposition (e.g., sputtering) exhibits another means of realizing metal coating, such as conductive LM-oxide/Pt composites [49]. These surface engineering methods can increase the conductivity of liquid metal particles when they are actually used as printing ink.

On the contrary, obvious interface feature transitions exist as oxidation increases with regard to the surface tension, mechanical strength, and wettability [52]. These transitions help LM to internalize additive materials, and thus possess excellent performance [53]. The typical approach is the mechanical stirring of LM in air to increase its oxidation, which may facilitate the uniform mixture of metal/metal or metal/nonmetal. Cao et al. mixed NdFeB microparticles with the LM matrix and then fully magnetized the composites through a strong impulse of the magnetic field [54] (Figure 3d). As the stirring time increased, the liquid-like suspension gradually changed into a solid-like composite, which presented unique electrical and magnetic responses (Figure 3e). Compositing other phase metal materials through the bulk internalization of liquid metal improves the conductivity and functionality of the bulk material. Recently, Hajalilou et al. systematically studied a series of biphasic LM composites [55], which combine the fluidity and self-healing properties of LMs, and the printability and elastic integrity of elastomers. In the selection and preparation of composite materials, more consideration should be given to the influence of the type, shape, and size of filler materials on the properties, microstructure, and even the bonding form between the filler and LM. The electromechanical coupling properties of these composites, in addition to their adhesion to the substrate and mechanical stability under external loading, have a large impact on the morphology and stability of printed electronics.

### 2.2. Liquid Metal-Ligand Molecule Composites

**Figure 4:**
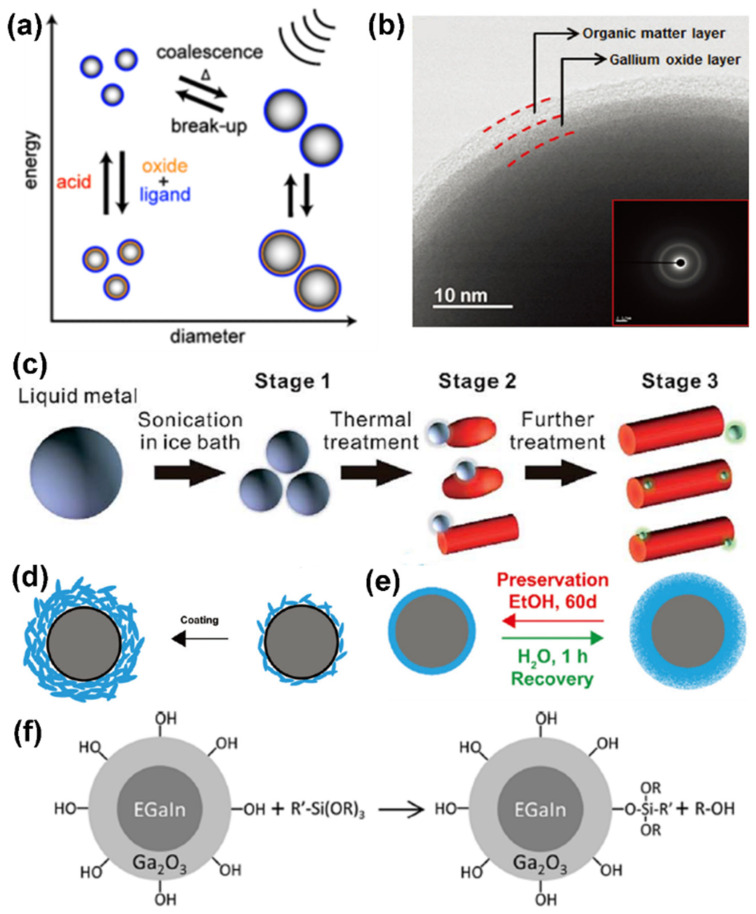
Liquid metal–ligand molecule composites. (**a**) Reversible size control of LM nanoparticles under ultrasonication. Reprinted with permission from Ref. [57] Copyright 2015 John Wiley and Sons. (**b**) HRTEM image of the bilayer of LM shell. Reprinted with permission from Ref. [58] Copyright 2016 John Wiley and Sons. (**c**) Illustration of the shape transform process under aqueous solution. Reprinted with permission from Ref. [60] Copyright 2017 Royal Society of Chemistry. (**d**) Scheme of degraded PVP molecules adsorbed onto the oxidized surface of LM nanoparticles. (**e**) Schematic illustration of the preservation recovery mechanism of LM–PVP nano-composites. Reprinted with permission from Ref. [61] Copyright 2020 Royal Society of Chemistry. (**f**) Scheme depicting the reaction of LM particles possessing hydroxylated gallium oxide shells. Reprinted with permission from Ref. [67] Copyright 2020 American Chemical Society.

The earliest metal inks were usually metal nanoparticles or metal nanowires dispersed in an appropriate solvent containing some specific additives to obtain a certain rheological behavior. This is conducive to the formation of uniform and continuous patterns during the printing process, and the attachment to the substrate. In order to fabricate functional devices by various printing techniques, the key step is to synthesize conductive LM-based nanomaterials. Because the ink should provide high conductivity of the printed pattern, it is essential that the conductive nanomaterials should have a high concentration and be non-aggregated and stable in the ink. The physical properties of the ink, such as viscosity and surface tension, are also critical to achieving high printing accuracy and resolution. From this point of view, formulations for printable liquid metals must prevent precipitation and aggregation. Although the presence of an oxide layer can maintain the colloidal structure and mechanical properties of LM [56], the shell is still too mechanically fragile and chemically sensitive to stabilize LM (especially particles). The homogeneous and colloidal LM nanoparticle suspensions are solution-processable and inkjet printable. The notable strategy for stabilizing and functionalizing LM particles is the assistance and decoration through ligand molecules. Under the excitation of ultrasonic energy, ligand molecules and LM particles undergo complex physicochemical transformation. Of course, the size and morphology of LM nanoparticles can be effectively regulated by selecting the appropriate ligand molecules and controlling ultrasonic parameters (time, power, temperature, ligand concentration, and pH) [57] (Figure 4a). Moreover, the LM–ligand molecules composites can still inherit the dispersibility, surface charge, surface functional groups, and even the surface activity of the ligand molecule. For instance, inspired by the adsorption of alkane thiols on gold’s surface, the sulfhydryl molecule-induced self-assembled monolayers (SAMs) on LM can compete with the new-birth oxide layer to suppress further oxidation [58]. The high-resolution transmission electron microscope (HRTEM) image (Figure 4b) indicates that the bilayer (gallium oxide layer and organic matter layer) structure can efficiently stabilize the LM particles. Nanoparticles with narrow distribution and stable dispersion in solvents can be obtained by subsequent centrifugation or filtration. The LM–thiol composites exhibit good dispersibility in alcohol (methanol, ethanol, and 2-propanol), which is the common solvent for sulfhydryl ligands. The processibility of LM–ligand molecule composites in aqueous solution will be greatly improved and broaden because water is a versatile and non-toxic solvent. Unfortunately, the existence of water and oxygen molecules can inhibit oxidation from pristine LM to GaOOH [59]. Simultaneously, the excessive temperature (>70 °C) in water will induce LM to generate a morphological transformation accompanied with a dealloying process [60] (Figure 4c). The suitable aqueous ligand must possess strong affinity to bonding with the LM surface and anti-oxidation ability. For example, the aqueous polymers and their derivatives, equipped with abundant hydroxyl, ether, carbonyl, and amine groups, can strongly combine with the metal/metal oxide (Ga/Ga^3+^) surface. Liu et al. utilized poly(vinyl pyrrolidone) (PVP) as a protective layer (up to 20 nm) to encapsulate LM nanoparticles [61] (Figure 4d). These LM–PVP composites are stable against long-term preservation in water up to 30 days and in ethanol up to 60 days. In addition, the PVP coating shows solvent responsive swelling/shrinking behaviors (Figure 4e). Similarly, polyvinyl alcohol (PVA) [62] was also used for uniformly dispersed LM particles, which came from the stable interaction between the hydroxyl group and the surface oxide layer. Recent work demonstrated that the phenolic compounds rich in catechol and pyrogallol groups can chelate the liquid metal surface and alleviate its oxidation. Ligand molecules such as dopamine [63], gallic acid [64], and tannic acid [65] may contribute to a strong coordination interaction between Ga^3+^ with catechol groups, which was established in iron-catechol chemistry [66]. It has to be mentioned that the siliconized LM can be obtained by the siliconization reagent reaction using the hydrogen bonds on the surface of gallium oxide [67] (Figure 4f). These LM–ligand molecule composites with high dispersion stability, sustainability, and functionality will provide sufficient convenience for large-scale processing and patterning.

## 3. The Printable Stretchable Electronics of LM-Based Nano-Composites

In the previous section, we introduced the synthesis of LM-based nano-composites; we continue to outline the targeted printing technologies compatible with inks and their characteristics below. Typically, printing techniques can be divided into two broad categories: non-contact patterning (or nozzle-based patterning) and contact-based patterning. The main difference between different printing technologies depends on the principle of printing the ink to the substrate. Non-contact technologies include inkjet printing, electrohydrodynamic (EHD) printing, and aerosol jet printing, whereas screen printing, gravure printing, and flexographic printing are examples of contact technologies. It is worth mentioning that due to the fluidity of liquid metal, it can be patterned and processed in ways that cannot be achieved with solid metal, such as 3D printing [68]. Here, we mainly highlight the recent progress made in planar patterning of LM-based printable electronics.

### 3.1. The Printing of LM-Based Nano-Composites

**Figure 5:**
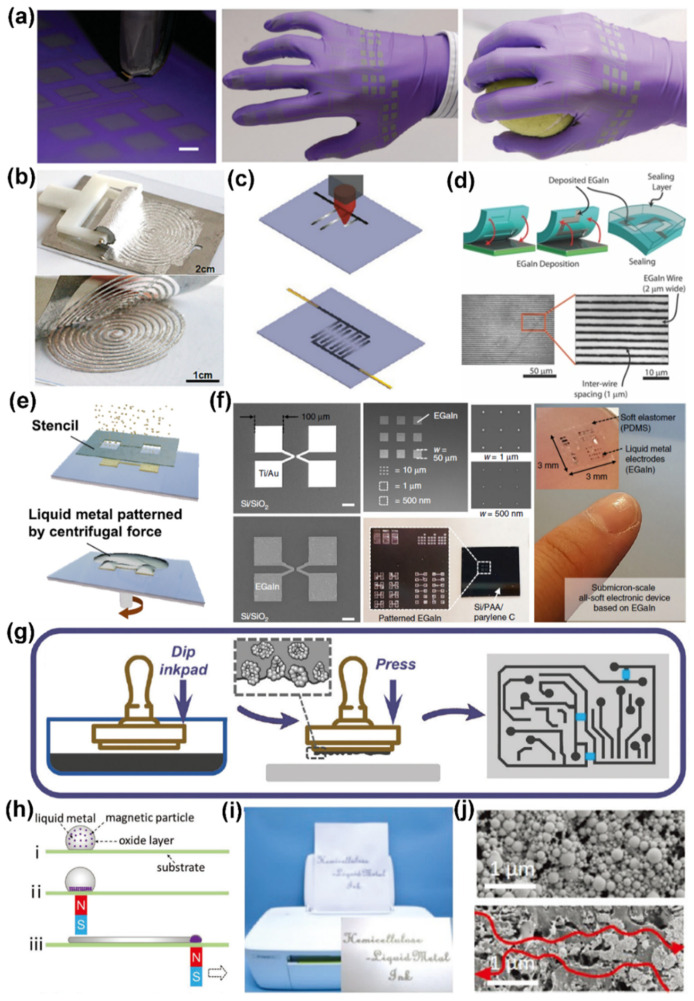
Printing methodologies of LM-based nano-composites. (**a**) Inkjet printing of LM nanoparticle-based inks. Reprinted with permission from Ref. [69] Copyright 2015 John Wiley and Sons. (**b**) The printing of Ni-LM on Eco-flex substrate via rolling brush. Reprinted with permission from Ref. [70] Copyright 2018 American Chemical Society. (**c**) The microcontact printing (μCP) of an LM-based circuit. Reprinted with permission from Ref. [72] Copyright 2018 American Chemical Society. (**d**) The deposition process of LM-based electronics with micro-scale line width. Reprinted with permission from Ref. [74] Copyright 2014 John Wiley and Sons. (**e**) The Stencil Lithography and Centrifugal Force-Assisted Patterning of Liquid Metal. Reprinted with permission from Ref. [75] Copyright 2021 American Chemical Society. (**f**) Nanofabrication process based on hybrid lithography for submicron-scale LM patterning. Reprinted with permission from Ref. [76] Copyright 2020 Springer Nature. (**g**) Schematic image of the procedure for printing based on conductive LM nano-clay. Reprinted with permission from Ref. [80] Copyright 2021 Royal Society of Chemistry. (**h**) Direct patterning of LM using a magnetic field. Reprinted with permission from Ref. [81] Copyright 2019 John Wiley and Sons. (**i**) Image showing the printing of DAX/LM nano-inks on paper using a commercial inkjet printer. (**j**) SEM images showing that the printing path can be activated by the erasing method. Reprinted with permission from Ref. [82] Copyright 2021 Royal Society of Chemistry.

For the purpose of soft and stretchable properties in electronics, there exist many challenges in developing LM-based printed devices that differ from conventional rigid electronics. To date, two dominant strategies of LM-based nano-composites patterning have been developed: (1) direct extrusion of stabilized LM composites (nozzle dispensing, ballpoint writing, and inkjet printing) and (2) deposition of the LM composites on substrate with a specific pattern (screen printing or transfer printing). Thus, the basic parameters for printable stretchable electronics are printing resolution, adhesion, conductivity, and deformability. Equipped with enhanced compositions, structures, and functional groups, the proposed LM nano-composites represent a great improvement in terms of conductivity, interfacial compatibility/wettability, and stability. In addition, coupled with its fluidity and self-healing ability, liquid metal-based nano-composites can be promising candidates for stretchable and elastic electronics. For example, the fluid properties allow direct injection and printing of liquid metal to form patterns using a variety of off-the-shelf equipment such as syringes and printers. These high-throughput and scalable processes enable direct inkjet printing of LM-based nano-inks onto elastomeric glove surfaces, forming strain gage arrays with complex wiring and contact pads [69] (Figure 5a). LM doped with various metallic or non-metallic materials can be designed to prepare ideal functional materials with tunable electrical, mechanical, and chemical properties, further expanding the processable range of LM-based nano-composites. The composite stirring of LM and Ni particles with different mass fractions can change its previous surface properties to obtain suitable adhesion and conductivity, and finally it can be directly brushed on the flexible substrate [70] (Figure 5b). The ability of liquid metal to flow freely through channels allows the design of reconfigurable devices. Utilizing the self-wetting property of LM in air, the printed route maintains the initially deposited feature shape during formation and encapsulation [71]. Tabatabai et al. incorporated microcontact printing and stamp lithography to produce LM circuits. In contrast to existing fabrication techniques, this extensible method can be used to produce circuits with any planar geometric feature, including electrodes with a large planar area (involving multiple intersections), intersecting and closed-loop wires, and combs with multiple terminal electrodes [72] (Figure 5c). There are still many challenges in printing methods to further reduce the feature size of liquid metal-printed traces to the micron level.

Due to the innovation in material systems and improvement in the printing technology, the printing characteristic parameters (line width, resolution) based on LM materials are constantly revealing new indicators [73]. Gozen et al. proposed an innovative strategy to fabricate high-density stretchable microelectronic devices with line widths and spacings as small as 2 and 1 μm, respectively [74]. As shown in the fabrication method illustrated in Figure 5d, the first step entails creating an elastomer mold with micron-scale concave features (microchannels), then using the roller to spread the LM on the pre-prepared mold. Finally, the LM-filled microchannels are sealed with an additional layer. Sun et al. introduced a facile but reliable process combing stencil lithography and centrifugal force assistance to realize micro-scale patterning of LM on an elastomer surface [75] (Figure 5e). Kim et al. presented a nano-fabrication strategy that combined electron-beam lithography for microstructures with soft lithography for pattern transfer. The approach enabled a high-resolution and high-density all-soft LM thin-film pattern with feature sizes as small as 180 nm and 1 μm line spacing [76] (Figure 5f). The printing methodologies of LM-based nano-composites also depend on the morphology and rheology of the ink, in addition to the dimensional requirements of the printed product (feature parameters, processing area, scalability, etc.). The semi-LM paste, gained from the mixture of LM and other metal, oxide, and magnetic additive elements, is a sticky viscous ink that can be easily printed for novel and complicated stretchable circuits [77,78,79]. For example, nano-clay was also used to improve the adhesion of LM [80]. The LM–clay mixture, having great conductivity, low electric hysteresis, and excellent damage mitigation ability, was accessible for in situ rapid printing (Figure 5g). Additionally, Ma et al. developed a versatile approach for direct patterning of LM using a magnetic field [81] (Figure 5h). These liquid metal-oxide/metal composites can further expand the composition system to fulfil the rheological requirements for universal printing methods without other post-processing. In addition, the ligand molecule-stabilized liquid metal nanoparticles can also be patterned through various techniques, including inkjet printing, direct writing, and dyeing. For example, Hao et al. utilized dialdehyde xylan (DAX) to fabricate sustainable, stable, and catalytic nano-inks for universal inkjet printing [82] (Figure 5i,j). The smaller the size of the ink, the higher the stability, and the line width of the printed electronic devices is reduced accordingly. Simultaneously, the stabilizing LM nano-ink having a low cost, long-term stability, biocompatibility, and reaction activity would enhance the stability and functionality of printed electronics. Nevertheless, the ligand-mediated nanoparticles are isolated from each other instead of being in contact to form a conductive pathway. These residual reagents on the surface of LM particles limit the conductivity of printed electronics to a certain extent. Additional post-processing procedures, usually defined as sintering, are essential to promote the coalescence of these particles [83]. For example, Li et al. applied tension or pressure to rupture the encapsulated oxide shell trace on the depositing paper, and the overflowing area of the LM spontaneously formed a highly conductive path [84]. In contrast with the mechanical sintering methods, the laser sintering methods can focus a laser beam to efficiently and precisely rupture and ablate LM particle oxide shells [85]. The initial non-conductive high-concentration LM–silicone (LMS) ink can be activated with freezing to form continuous conductive networks [86].

**Figure 6:**
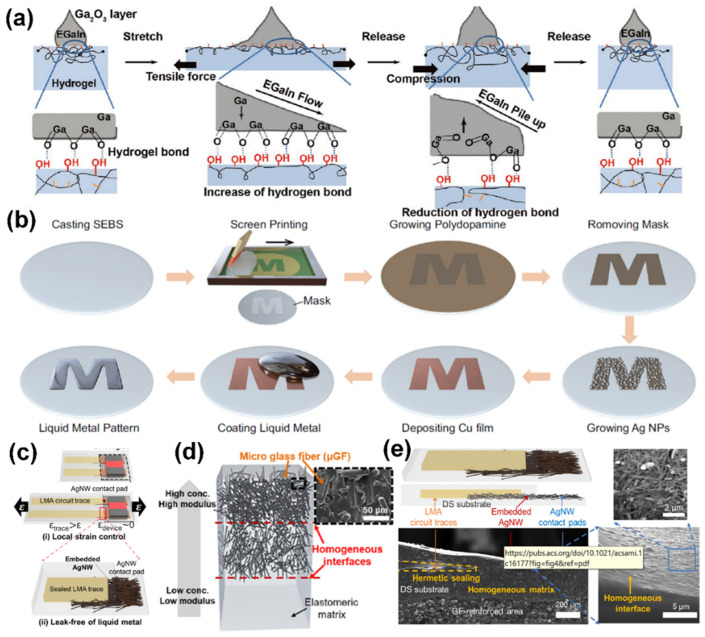
The selected printing of LM-based nano-composites. (**a**) Photographs and illustrations of LM droplets’ surface reconciliation on hydrogel during stretching and releasing. Reprinted with permission from Ref. [87] Copyright 2020 John Wiley and Sons. (**b**) The process to create a specific LM pattern on elastomer. Reprinted with permission from Ref. [35] Copyright 2021 Springer Nature. (**c**) Unit structure of the LM-based circuit including two design strategies for reliability enhancement. (**d**) The homogeneous interfaces between the matrices with different elastic moduli. (**e**) The leak-free structure of embedded AgNW networks. Reprinted with permission from Ref. [88] Copyright 2022 American Chemical Society.

LM-based printed electronics exhibit an excellent combination of electrical conductivity and stretchability, and lower electrical hysteresis and infiltration network degradation, than rigid materials under strain. However, the high surface tension and oxidation of gallium in air results in great challenges to high-resolution LM patterning. In order to further improve the printing quality and simplify pattern process, researchers have designed many ingenious printing strategies and structures that can effectively enhance the accuracy of the printing trajectory and the bonding force of the printing interface. It is noteworthy that the selective wettability is directly related to pattern uniformity and resolution. Poor wetting will make the LM difficult to extrude, or produce features with poor accuracy and repeatability, resulting in inconsistent print and placement positions. Silva et al. realized selective wetting of printing circuits on PVA-coated substrates [18]. In addition, the interactions between numerous hydroxyl groups (-OH) in hydrogel and the native gallium oxide (Ga_2_O_3_) can also spread LM droplets [87]. The micropatterned LM droplets can autonomously reconciliate their surface to a hydrogel for a continuously conductive interface having 1500% elongation (Figure 6a). Well-defined pattern features for liquid metal can be directly placed on the substrate in one step by selective wetting. As shown in the process in Figure 6b, Zhu et al. adopted screen printing to define the locations and morphologies as desired pattern features on the elastomer substrate [35]. Then, they deposited a layer of polydopamine (PDA) as the self-polymerization initiating layer to realize surface functionalization, which can also subsequently facilitate deposition of Cu film as a LM-reactive wetting layer. The patterned LM-based circuits exhibit high resolution (100 μm), excellent electrical conductivity (4.15 × 10^4^ S $m^{-1}$), and ultrahigh stretchability (1000% tensile strain). However, in conventional LM-based stretchable electronics, the obvious obstacle of liquid leakage exists, which may result in performance deterioration and, ultimately, electrical failure. Leakage usually occurs at junctions between adjacent materials having different mechanical properties, i.e., heterogeneous interfaces between soft polymer substrates and rigid electrodes, because the weak bonding is unable to withstand stresses resulting from external deformations. Kim et al. proposed a printing scheme to realize reliability enhancement by local strain control and a leak-free design [88] (Figure 6c). In order to reduce the interface heterogeneity of the printing layer, a glass fiber reinforcement (GFR)-based modulus-gradient structure was employed in the stretchable substrate to satisfy intra-structural and external device mounting stability (Figure 6d). The embedded AgNW-elastomer composite structures can hermetically seal the LM, resulting in a leakage-free characteristic (Figure 6e). Due to the transition interface from rigid to flexible, the device does not experience signal attenuation under external stress stimulation.

### 3.2. The Application of Printable LM-Based Stretchable Electronics

LM-based stretchable electronics are widespread in human–machine interfaces (HMIs), wearable health monitors, and soft robotics. Printing methodologies allow for efficient production of customizable and scalable function electronics (sensors, conductors, actuators, etc.). More importantly, the electrical response of LM-based stretchable electronics is critical to practical application.

High-performance stretchable conductors (electrodes) are critical to offering stretchability and conductivity [89]. Due to the natural skin affinity of LM, printed LM-based stretchable conductors are highly desirable for precise electric signal transmission in smart epidermal electronics. Ma et al. reported a stretchable conductor fabricated by printing liquid metal onto an electro-spun elastomeric fibrous mat [9]. Benefiting from the in situ binding of the LM droplets with the lateral and vertical mesh-like fiber structures, the stretchable conductor simultaneously offered high permeability in air, stretchability (over 1800%), conductivity (1.8 $\times \;10^{6}$ S $m^{-1}$), and electrical stability (washable). The repeated electrospinning and printing processes were performed to fabricate vertically stacked multilayer electrical circuits for monitoring electrocardiography (ECG) signals in daily life (Figure 6a,b). The stretchability and mechanical durability of the liquid metal nano-composite-based conductors were significantly improved compared to earlier reported elastic conductors. During stretching, the encapsulated EGaIn is released from the broken Ga_2_O_3_ shells, which preserve the continuous electrical interconnections. In addition, the biphasic pattern of liquid metal composites can spatially regulate electrical transformation behavior caused by the unique interface between liquid metal and substrates [90]. The hard segment between the soft liquid metal and substrates can assume the role of stress concentration.

Simultaneously, the LM-based printed stretchable sensors can be exploited to monitor human electrophysiological signals [91]. The spherical structure of LM shows an obvious mechanically induced electrical response, which display a series of novel phenomena under strain. According to Ohm’s law, the resistance (R) of a conductor is related to its length (L) and resistivity (ρ), and is directly proportional to its cross-sectional area (S) and inversely proportional to it. Thus, most elastic composites exhibit a negative piezo-conductive effect, which means the conductivity decreases under tensile strain. However, the leakage of EGaIn will maintain the conductive pathway under large deformation. The LM-based composites usually exhibit a positive piezo-conductive effect and show a unique transformation in the mechanical and electrical change curve, and can thus be fully developed and designed as stretchable sensors [92]. Gao et al. reported a microfluidic tactile sensor based on a diaphragm pressure sensor design [93]. The LM droplets in the microchannels rupture and flow along the channel paths under the deformation of the elastomer. The dynamic resistance change can be utilized to monitor human health and reflect activity postures (Figure 6c–e). Simultaneously, every LM droplet can be regarded as a “cistern of electricity”, which can easily affect the resistance transition of the whole device. By introducing “liquid metal cells” and “liquid metal nerve endings” as the basic sensing unit, the printed LM films can feasibly be used to fabricate several sensing arrays (Figure 6f). Li et al. utilized the patterned conductive films as artificial sensory systems to simulate the mechanism of pain sensitization before and after a nerve ending injury [94]. Concurrently, the LM-based printed stretchable sensors have been used to realize pressure, breath, and temperature-stress bimodal sensing [95,96,97].

**Figure 7:**
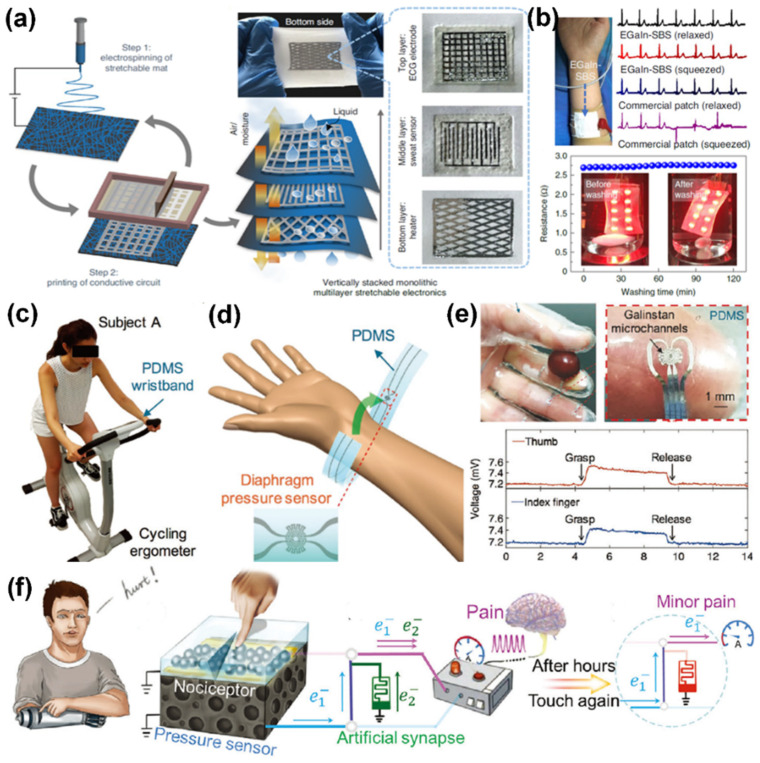
The printable LM-based stretchable electronics for signal detection. (**a**) The fabrication of the vertically stacked monolithic stretchable mat by alternating electrospinning of SBS fibers and stencil printing of LM electrodes. (**b**) The ECG detection of the three-layer monolithic stretchable device at different strains in response to different sweat volumes (phosphate-buffered saline (PBS) was used to represent sweat and the washable reliability). Reprinted with permission from Ref. [9] Copyright 2021 Springer Nature. (**c**) Optical image of a subject wearing the PDMS sensor wristband on a cycling ergometer. (**d**) Schematic of how the sensor is worn for measurements. (**e**) The tactile sensing glove worn while grasping a grape and the real-time response. Reprinted with permission from Ref. [93] Copyright 2017 John Wiley and Sons. (**f**) The bio-inspired multi-mode pain-perceptual system. Reprinted with permission from Ref. [94] Copyright 2021 John Wiley and Sons.

**Figure 8:**
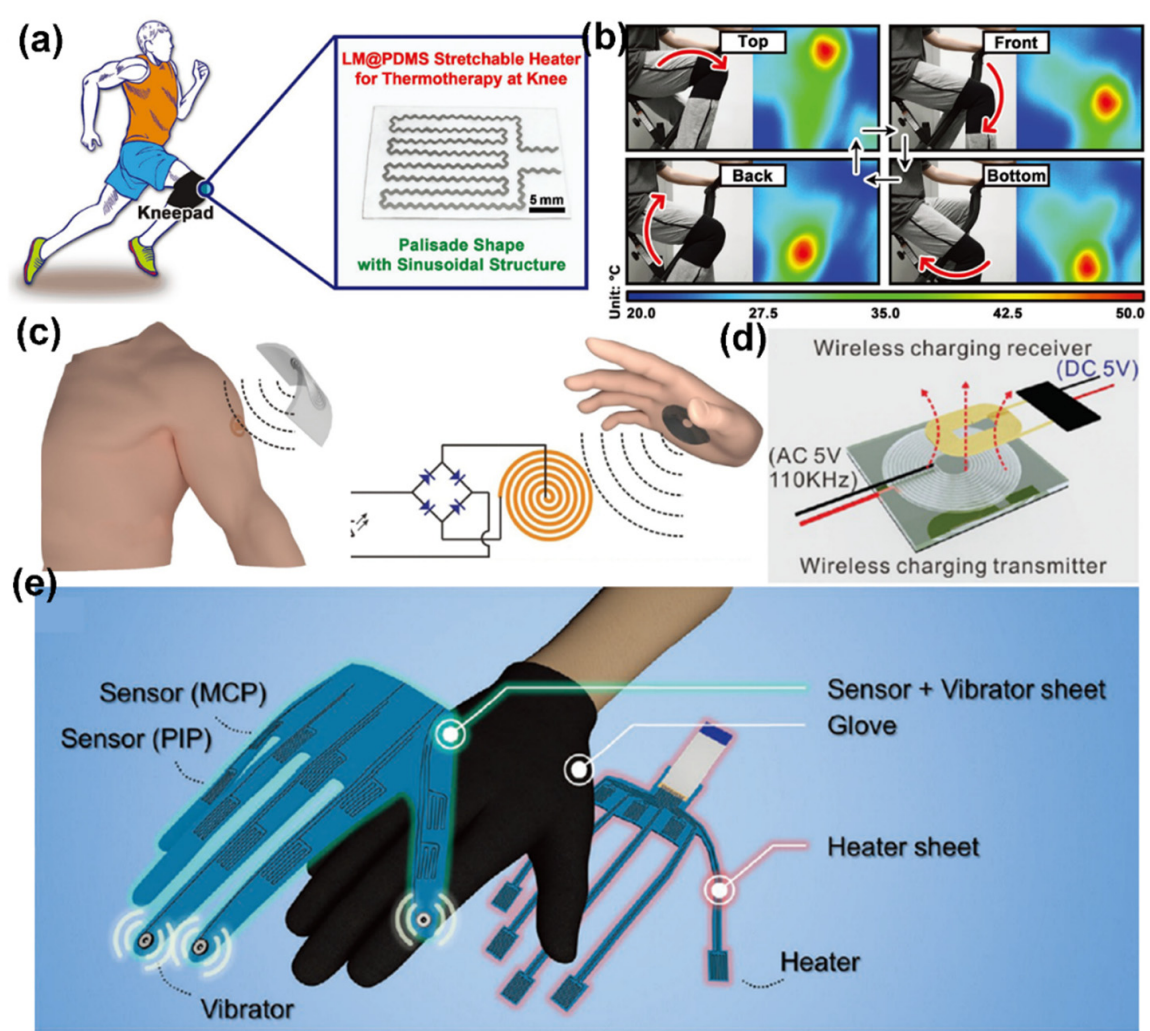
The wearable devices of LM-based printed stretchable electronics. (**a**) Optical photo of specially designed LM@PDMS stretchable heater and schematic illustration of its working condition. (**b**) Optical photos of exercise at different states and corresponding IR thermal images. Reprinted with permission from Ref. [99] Copyright 2019 John Wiley and Sons. (**c**) Implementation of the flexible transmit coil into the palm location to power the wireless receiver. (**d**) Principle of wireless charging via inductive coupling. Reprinted with permission from Ref. [101] Copyright 2019 Royal Society of Chemistry. (**e**) An exploded view of the multimodal sensing and feedback glove. Reprinted with permission from Ref. [40] Copyright 2021 John Wiley and Sons.

Concurrently, printed electronics are entering the consumer electronics market at a rapid rate, including in personal thermal management and wearable health-care devices [98]. For example, Wang et al. used the conductive composite of LM and polydimethylsiloxane (PDMS) as ink to directly print a flexible heater having a sinusoidal structure (Figure 7a) [99]. The printed LM@PDMS heater displays instant heating temperature response and relatively low temperature variation (8%) at the large strain levels (100%), which indicates a great potential in wearable thermotherapy (Figure 7b). Recently, the development trend is for wearable electronics to be wireless and self-powered, and to be operated conveniently for a long period, and thus to be easily compatible with electric vehicles, drones, smart home systems, and implantable medical devices [100]. Teng et al. utilized the printed LM conductor as transmitting and receiving coils for wireless power charging (WPC) (Figure 8c,d) [101]. Zheng et al. used the highly conductive Ag@LMPs as the conductive ink to fabricate NFC antennas by screen-printing for reading a web address [26]. The smart wearable device can be regarded as technology for virtual reality (VR), which can easily realize an immersive contact-free enhancement. The LM can be directly printed in the sensor sheet of database gloves to measure finger movement and provide vibro-haptic feedback under stretched conditions (Figure 8e) [40]. The printable stretchable electronics of LM-based nano-composites will provide new opportunities, from concept to business.

## 4. Summary and Outlook

Liquid metals (particularly gallium and its alloys) have the characteristics of fluidity, conductivity, stimuli responsivity, and chemical reactivity. The intrinsically generated oxide layer on LM endows the unique morphological characteristics and transformation principle. In this review, the basic synthesis methods of liquid metal-based nano-composites were summarized as solutions for printable materials. The LM-based composites constructed with other materials (metal, oxide metal, ligand molecules) will significantly enhance the processibility and functionality of printable stretchable electronics. First, the resulting semi-paste LM composites can efficiently meet the requirements of printing. Second, nano-composites will present multiple functions due to the introduction of additives and the sufficient adhesion to substrates resulting from ligand stabilization. For the next generation of LM-based composites, the dilemma between stability and conductivity should be expertly addressed; that is, the strategies to achieve anti-oxidation and initial conductive properties should be further studied and developed. A new “library” of ligand molecules should be established to prevent the inevitable oxidation of Ga in air. In addition, more fabricating or printing methods are highly desirable to gain initial conductivity without external sintering. It is necessary to develop new material systems and develop new dispersion technologies to improve the uniformity of LM-based composite materials. The optimized solutions will clearly contribute to the expansion of LM-based applications in delicate controllable LM pastes, precise zero-loss circuits, artificial perceptual interfaces, and novel systems for the Internet of Things.
